# Optimization of the Sustainable Production of Resistant Starch in Rice Bran and Evaluation of Its Physicochemical and Technological Properties

**DOI:** 10.3390/polym14173662

**Published:** 2022-09-03

**Authors:** Ruta Vaitkeviciene, Joana Bendoraitiene, Rimgaile Degutyte, Mantas Svazas, Daiva Zadeike

**Affiliations:** 1 Department of Food Science and Technology, Faculty of Chemical Technology, Kaunas University of Technology, 50254 Kaunas, Lithuania; 2 Department of Polymer Chemistry and Technology, Faculty of Chemical Technology, Kaunas University of Technology, 50254 Kaunas, Lithuania

**Keywords:** rice bran, ultrasound cavitation, resistant starch microstructure, crystallinity, hydration properties, mechanical performance

## Abstract

In this study, the optimization of ultrasound (US) (850 kHz, 120 W) processing parameters (temperature, time, and power) for the enhanced production of resistant starch (RS) in rice bran (RB) matrixes was performed. The effect of US cavitation at different temperatures on the morphology, physicochemical properties, and mechanical performance of RS was evaluated. Ultrasonication at 40–70 °C temperatures affected the chemical structure, reduced the crystallinity of RS from 23.85% to between 18.37 and 4.43%, and increased the mechanical and thermal stability of RS pastes, indicating a higher tendency to retrograde. US treatment significantly (*p *< 0.05) improved the oil (OAC) and water (WAC) absorption capacities, swelling power (SP), solubility (WS), and reduced the least-gelation concentration (LGC). The mathematical evaluation of the data indicated a significant effect (*p* < 0.05) of the US parameters on the production of RS. The largest increment of RS (13.46 g/100 g dw) was achieved with US cavitation at 1.8 W/cm^2^ power, 40.2 °C temperature, and 18 min of processing time. The developed method and technology bring low-temperature US processing of rice milling waste to create a new sustainable food system based on modified rice bran biopolymers.

## 1. Introduction

Cereal processing by-products, being a low-cost source of nutritionally valuable components, such as dietary fibres, resistant starches, proteins, minerals, and bioactive compounds, are promising materials for the valorization of sustainable bio-based components for food.

Resistant starch (RS) is a biopolymer of plant origin that is resistant to digestion in the small intestine [1]. In terms of RS’s chemical and structural features, five types of RS have been identified [2]. RS1 is a starch that is physically unavailable for digestion by enzymes due to its intact seed or grain structure. RS2 refers to the inherent resistance of starch granules (potato, banana, etc.) to digestion due to its native conformation. RS3 represents the largest RS fraction, which is primarily retrograded amylose. RS type 4 is a chemically modified starch, and RS5 is a helical-structured lipid–amylose complex.

Starch is composed of the two polysaccharides, amylose, a linear polysaccharide with α-1,4-glycosidic bonds, and amylopectin, which is a larger branched polysaccharide with α-1,4 and α-1,6 linkages [3,4]. The crystalline region consists of double helices of amylopectin, while the amorphous region is formed by amylose chains and branched segments of amylopectin. Starch retrogradation involves the recrystallization of amylose chains and the formation of tightly packed double helices stabilized by hydrogen bonds, and by the association of amylopectin chains into double helices, it is thus protected from enzyme attack [3]. Retrograded starch can seriously affect the functional properties of starch products, such as texture, stability, transparency, viscoelasticity, and digestibility [4]. The factors affecting starch retrogradation include the structure and the type of starch, the amylose to amylopectin ratio, storage conditions, and the starch modification process [4,5,6].

Nowadays, RS is characterized as a functional biopolymer with physiological effects comparable to those assigned to dietary fiber [2]. RS also brings a positive nutritional effect to food products as a functional ingredient and an exceptional prebiotic [7,8].

Rice processing by-products have the potential to be utilized as an RS source, but the natural RS content depends on the rice variety and can be relatively low [9]. Moreover, the better part of the conventional processes of food production decreases the undigestible fraction of starch in foods [7]. The content of RS can be enhanced during technological processes [10,11], and the physical or hydrothermal modification of starchy raw material [12,13]. The emerging food processing technologies, effectively changing the physicochemical properties and mechanical performance of food components, are of great interest nowadays [14,15].

The nutritional and technological properties of foods can be maintained through the use of preserving production conditions, leading to the increased interest in developing safe and cost-effective technologies. In the last decade, ultrasound (US) has been used extensively in food processing to enhance the physicochemical and functional properties of food components [16,17,18,19,20]. However, most of the research so far has focused on systems based on pure starch [21,22], although starch as such is not consumed. Future research, therefore, should be directed to RS within the context of an all-food matrix.

The technological and mechanical properties of starch depend on water absorption and starch formulation, processing, and retrogradation. Higher amylose/amylopectin ratios of starch generally give material of higher strength and plasticity, and also affects the gelatinization process, which generates the uniform amorphous thermoplastic structure by the action of temperature and mechanical force or ultrasound cavitation [14,23]. Quantifying these variables is important for the use of starch materials in different applications.

From this perspective, our work has been devoted to the development of a new sustainable food system based on rice bran biopolymers, in light of the potential application of US technology for the enhanced production of RS, by optimizing RS3 preparation conditions, thereby taking into account the effect of US processing temperature on the morphology, structural changes, mechanical performance, and technological properties of RS, and the possibility of the valorization and recovery of rice milling by-products.

## 2. Materials and Methods

### 2.1. Rice Bran Material

In this study, the analyzed food matrix was rice bran (RB) obtained from a local mill (SC “Ustukiu malunas”, Pasvalys, Lithuania) after the brown rice milling process. A fraction with a particle size of 315 µm (protein 11.20%, starch 66.41%, carbohydrates 81.53%, fat 5.38%, ash 1.89% dry wight basis, dw) was used for the experiment. Commercial native rice starch (S-7260) was purchased from Sigma Aldrich (Saint Louis, MO, USA). Raw materials were stored in plastic jars at 4 °C temperature.

### 2.2. Ultrasonication Procedure

Parameters of the starch sonication were selected on the basis of procedures reported by Wang and Bai [24]. For the US treatment, the RB sample (10 g) and distilled water were mixed at a solid/liquid ratio of 1:3. The mixture was placed in a tightly closed plastic bag (sample thickness 15 mm), and the sample was treated with US in an ultrasonic high-power bath (Type 5/1575) connected to a 120W-HF-Generator (Meinhardt Ultraschalltechnik, Leipzig, Germany). Sonication was carried out at 850 kHz frequency and 50% US intensity for different times (15–35 min) at different temperatures (30–70 °C). After sonication, each sample was subsequently stored at 4 °C for 24 h, then dried by an oven at 50 °C and subjected to the isolation and determination of the starch. Each experiment was repeated four times.

### 2.3. Starch Content Measurement

The content of resistant starch (RS) was determined using a Megazyme Assay Kit (K-RSTAR, Megazyme Int., Wicklow, Ireland) for resistant starch determination based on the AOAC method (2002.02). The RB sample (100 ± 5 mg) was digested with a pancreatic α-amylase and amyloglucosidase (PAA/AMG) at 37 °C (pH 6.0) for 16 h with continual stirring, during which time the non-resistant starch was solubilized and hydrolyzed to D-glucose. The reaction was terminated by the addition of an equal volume of 95% ethanol and RS was recovered as a pellet through centrifugation (3000× *g*, 10 min); this was then washed twice with 50% (*v*/*v*) ethanol and centrifuged. RS in the pellet was dissolved in 2M KOH by vigorously stirring for 20 min in an ice-water bath. The solution was neutralized with acetate buffer and the starch was quantitatively hydrolyzed to glucose with AMG. D-Glucose was quantified with glucose oxidase/peroxidase reagent (GOPOD), and this was the measurement of the RS content in the sample. Digestible starch (DS) was determined by measuring free-glucose content with GOPOD in the original supernatant and the washings. The RS and rapid digestible starch (RDS) contents were calculated and expressed as grams per 100 g of the dry weight of raw material.

### 2.4. Amylose Content Determination

The amylose content in the RS samples was determined using a Megazyme Amylose/Amylopectin assay kit (Megazyme Ltd., Winklow, Ireland) according to the manufacturer instructions.

### 2.5. Starch Isolation

Starches were isolated from the RB material by the alkaline extraction before and after the enzymatic digestion step [10]. The starch isolation scheme is presented in Figure S1. The procedure of the isolation of starch comprises the mixing with 0.2% NaOH for 2 h and further centrifugation at 3200× *g* for 15 min. The extraction was repeated two times. After the protein extraction, the starch precipitates were washed two times with 150 mL of distilled water by centrifugation. Isolated starches were lyophilized and stored in a desiccator until further use.

### 2.6. Starch Morphology Evaluation

Granule morphology of starches was quantified using a scanning electron microscopy (SEM) (Quanta 200 FEG, FEI, Hillsboro, OR, USA). Each powdered sample was placed on an adhesive tape attached to an aluminum stub and randomly examined at 2500× magnification.

### 2.7. Crystallinity Analysis

Starch crystallinity was analyzed with a D8 Advance X-ray Diffractometer Bruker AXS (Bruker, Karlsruhe, Germany). Data were collected using CuK_α_ radiation (U = 40 kV and I = 40 mA), a 0.02 mm nickel filter, the Bragg-Brentano geometry, and a fast-counting detector Bruker LynxEye. The samples were scanned over the 2θ range of 5–30° at a speed of 6°/min. The relative crystallinity was determined by calculating the areas below and above the curve that correspond to the amorphous and crystalline regions, respectively [25]. The ratio of the amorphous area to the total area was taken as the relative crystallinity.

### 2.8. Fourier Transform Infrared Spectroscopy (FT-IR)

The FT-IR spectra of the samples were recorded using an FT-IR Spectrometer (Frontier, PerkinElmer Inc., Waltham, MA, USA) with a single reflectance horizontal ATR (Attenuated Total Reflectance) cell equipped with a diamond crystal. The data were recorded in the range from 4000 to 560 cm^–1^ with a resolution of 4 cm^–1^.

### 2.9. Pasting Properties

The thermomechanical behavior of the RS paste (peak viscosity, pasting temperature, final viscosity) was analyzed by a Brabender Micro-Visco-Amylograph (Brabender GmbH, Duisburg, Germany) according to Yang et al. [18] with some modifications. Suspensions of each sample (10%, *w*/*w*) were heated from 25 to 95 °C at a rate of 6.5 °C/min, held at 95 °C for 15 min, cooled to 50 °C at 6.5 °C/min, and held at 50 °C for 2 min with a speed of rotating paddle of 250 rpm.

### 2.10. Specific Viscosity Measurement

The mechanical properties of RS slurry (0.4 mg/mL) in 1N KOH were investigated by an Ostwald’s capillary viscometer (tube diameter 0.99 mm) held in a temperature-controlled water bath (Bath-Immersion Thermostat E5, medingLab Temperiertechnik, Hochdorf, Germany) [26]. Time of flow was measured four times, and the average values were taken for specific viscosity calculation.

### 2.11. Least Gelation Concentration

The least-gelation concentration (LGC) of starches was determined according to the method of Sathe and Salunkhe [27], analyzing the ability of starch suspension to form a gel after heating and cooling. For the experiment, test tubes containing 5 mL of 2–20% starch suspensions in distilled water were heated in an 80 °C water bath for 1 h, followed by rapid cooling in an ice-water bath, and further cooling at 4 °C for 2 h. The LGC was defined as the concentration allowing the gel to not slip from the tubes when inverted.

### 2.12. Experimental Design and Statistical Analysis

A Central Composite Design (CCD) consisting of 21 experiments and 3 center points for each power group was employed as the experimental design performed in a random order at four replications. The factors investigated included independent variables: temperature (*T*, °C), treatment time (*t*, min), and power (*P*, W/cm^2^) at different levels. Response Surface Methodology (RSM) [28] was used to investigate the effect of the US-assisted treatment parameters on the resistant starch (RS) content. Furthermore, the optimization of US processing conditions was performed by developing the simplest possible mathematical model with a coefficient of determination higher than 80%. The response was the RS content exclusively obtained under the influence of US action (dY, g/100 g dw). Results were analyzed using Design-Expert trial version 13.0.1.0 software (StatEase Inc., Minneapolis, MN, USA). The analysis of variance (ANOVA) was conducted for the assessment of the suitability of the model using the coefficient of ‘*lack of fit*’ and the Fisher value (*F*).

All chemical analyses were performed at least in triplicate. Statistical analysis of the data was performed using SPSS software (ver. 27.0, IBM, Chicago, IL, USA). The significant differences between the means were evaluated by one-way ANOVA at a significance level of 0.05.

## 3. Results

### 3.1. Resistant Starch Production Rate

**Figure 1:**
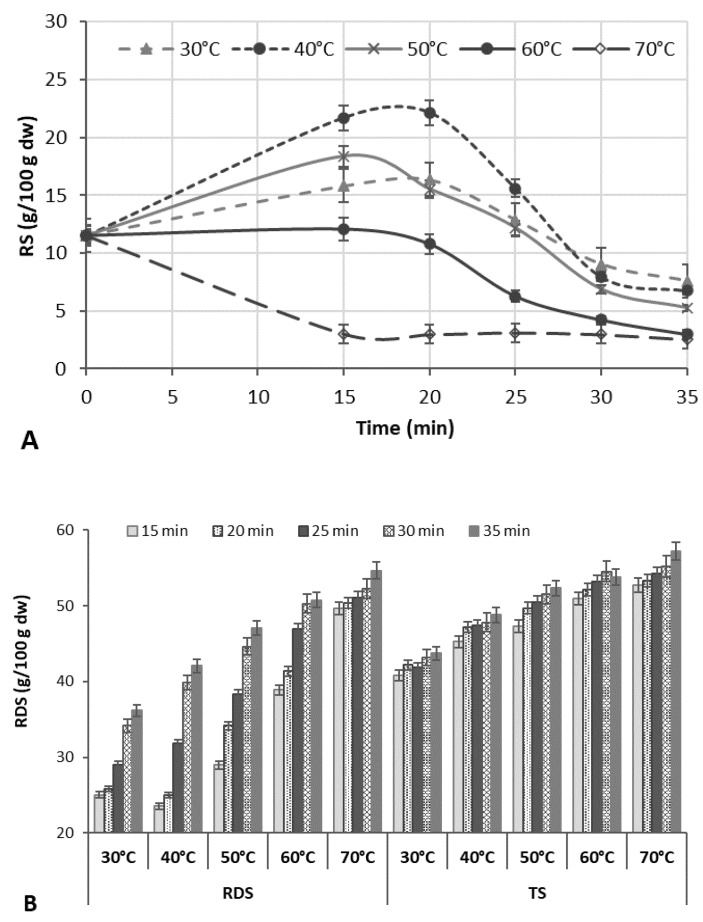
The effect of rice bran (*m/w* ratio 1:3) ultrasound treatment at different temperatures and times on resistant starch (RS) (**A**), rapid digestible starch (RDS), and total starch (TS) (**B**) contents.

Factors affecting the increment of RS due to the formation of RS type 3 in the rice bran matrix were studied at 50% US intensity (Figure 1A). The amount of RS3 measured at different US processing time points was compared to the initial content in the untreated RB (11.51 g/100 g d.w.).

Most of the RS was produced at the early period of ultrasonication (15–20 min); a longer US treatment time (25–35 min) enhanced the degradation of starch, significantly decreasing the content of RS. Sonication at higher temperatures (60–70 °C) caused a slight increase (up to 5.0%) in the RS content during 15–20 min of ultrasonication, but a decrease up to 73.5% at longer sonication times (25–35 min) was fixed (Figure 1A). Herewith, the rapidly digestible starch (RDS) content increased by 22.7–53.4% with increasing US time and temperature, increasing the total starch (TS) extraction yield (Figure 1B).

The results are in agreement with other reports, showing that the yield of starch could be improved due to acoustic cavitation that disrupts the protein and fibre matrix, allowing the enhanced release of the starch granules [29]. In other cases, the US-initiated increase in the RS content at US temperatures of 40–50 °C may be due to the increased amylose content because of the cleavage of long chains and depolymerization of amylopectin due to the effect of US cavitation, contributing to the alignment or aggregation of the molecule, which leads to an enhanced yield of retrograded starch [18].

According to the literature [30], the US pre-treatment destroyed the amorphous areas of rice starch granules with a low impact on the crystalline pattern and chain length distribution of amylopectin [30]. Debranched starches can be involved in the formation of RS3, since the linear fragments of amylose with 13–30 glucose units of greater mobility and long amylopectin chains strongly contribute to RS3 formation [30]. Reduced chains of amylose have a tendency to form a more crystalline strongly bonded structure with a higher concentration of more stable and resistant double helices. The release of amylopectin side chains before retrogradation favored re-association leading to reduced starch digestibility [31].

At US temperatures above the rice starch gelatinization temperature, the layered structure of the starch became unstable, and that may be the reason for the reduced relative crystallinity and lower content of RS. The ANOVA of the initial experimental data showed that both the US temperature and time were the significant factors (*p* < 0.0001) for the RS production (Tables S1 and S2). The RS contents in all treatments at 30–50 °C significantly depended on the temperature (*p* = 0.0001, F = 267.36–749.01), and time (*p* = 0.0001, F = 21.63–576.92).

### 3.2. The Effect of Ultrasonication on Resistant Starch Increment and Mathematical Model Analysis

The effect of the US processing temperature (*T*), time (*t*), and power (*P*) on the RS yield was determined via optimization by CCD and RSM. The yield of RS varied significantly from 1.51 to 12.61 g/100 g d.w. when applying different *T* (30–50 °C), *t* (15–25 min), and *P* (1.3, 1.5, and 1.8 W/cm^2^) combinations (Table 1).

A quartic-order regression model (Table S3), describing the relationship between the RS content increment (dY = Y − Y_0_), obtained under the influence of the US processing, and independent variables, was constructed in the following equation:

dY = Y − Y_0_ = 11.65 − 3.33*t* − 0.5519*T* + 0.9644*P* − 0.8837*tT* − 0.28*tP* − 0.1419*TP* − 3.6*t*^2^ − 6.27*T*^2^ − 0.1387*tTP* + 0.8807*t*^2^*T* − 0.1393*t*^2^*P* + 1.19*tT*^2^ − 0.023*T*^2^*P* + 2.47*t*^2^*T*^2^ – 0.0643*t*^2^*TP* + 0.3412*tT*^2^*P*, (1)

where dY is the RS content increment; Y is the total RS content; Y_0_ is the RS content in raw material; *t, T*, and *P* are the values of temperature, time, and US power, respectively.

The ANOVA of the model confirmed that the sonication temperature, time, power, and their quadratic effects and linear interaction effects among the factors *t* and *T* significantly affected the RS formation (*p* < 0.0001) (Table S4). In addition, there were significant linear interaction effects among the factors of *tP* and *TP* (*p* = 0.0002 and *p* = 0.0094, respectively), and their quadratic effects on the RS content: *T*^2^ (F = 13636.80) > *t* (F = 5168.84) > *t*^2^ (F = 4055.14) > *P* (F = 1049.64) > *T*^2^*t*^2^ (F = 1001.51) > *Tt* (F = 728.10) > *tT*^2^ (F = 437.29) > *t*^2^*T* (F = 263.98) > *T* (F = 163.30).

Based on the statistical analysis, the model was significant for dY, showing that it was reasonably reproducible and well-fitting to the experimental data: Fisher value—2839.52 (*p* < 0.0001), variation coefficient 1.19 %, determination coefficient R^2^ = 0.9998. The results showed that the *p*-value for ‘*lack-of-fit*’ was not significant (*p* = 0.1049); hence, the model was satisfactory in explaining the obtained data at a 95% confidence level. The predicted R^2^ value (0.9736) for the experimental design was close to the adjusted R^2^ value (0.9994), indicating that 97% of the data could be described by this regression model. Comparing the experimental data with the theoretical prediction, the relative error (RE) varied between 0.17 and 1.56% (Table 1), indicating that the model could be used to estimate the response for the optimization.

To our knowledge, there are no reports on the optimization of high-power US-assisted production of RS in rice bran. The optimization of the US process conditions to increase the RS content was previously demonstrated for pure pea starch and buckwheat starch as raw materials [24,32]. However, such process could have complicated applications on an industrial scale because of the high cost of pure starches. In the present work, the US processing was optimized by implication of a different approach, and rice milling by-products were used as the raw material instead of pure rice starch to withdraw the losses of bran nutrients while improving the RS content; these results can be preferable for the food industry.

### 3.3. Optimization and Prediction of Process Parameters

**Figure 2:**
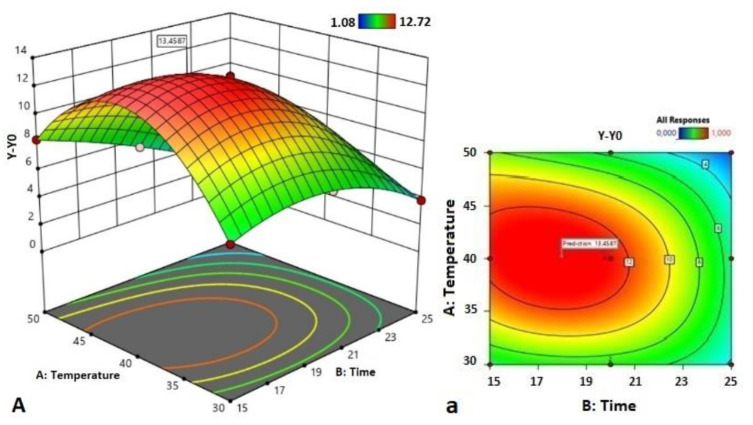

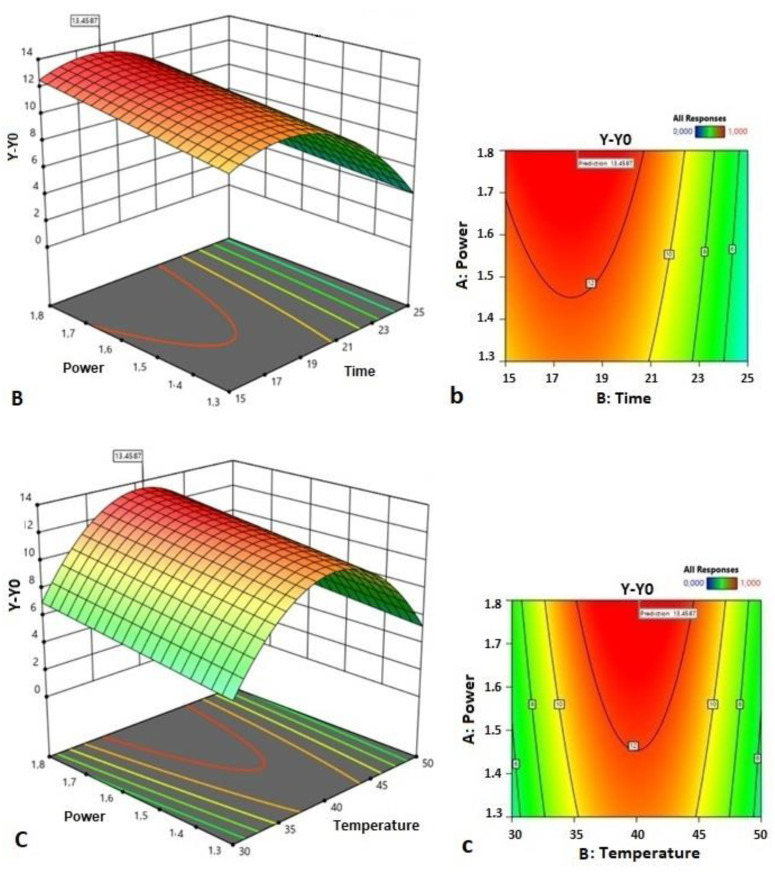
Response surface plots (**A**–**C**) and contour plots (**a**–**c**) of the interactions between ultrasound temperature and time (**A**), power and time (**B**), and power and temperature (**C**) for the RS increment (dY = Y − Y_0_).

Based on the obtained model, a three-dimensional plot was constructed to predict the relationship between the independent variables. Predictive plotting of the optimum conditions based on the increase in RS content was carried out by varying the factors. Figure 2 presents the relationships between the response value (dY) and the independent variables (time, temperature, and power).

The elliptical form of the contours and a relatively sharp slope of the surface responses indicate a strong interaction between the analyzed factors and a strong influence of the sonication parameters on the response value. Based on RSM, the optimal conditions for the production of RS in RB were set as follows: US power of 1.8 W/cm^2^, temperature 40.2 °C, and time 18 min. However, the optimal conditions proposed for the US power were located at the maximum level studied (+1), indicating that there is a possibility of exploring the production of RS outside the studied level, but this would be outside of the technical characteristics of the device used.

Under optimal conditions, the predicted RS increment (dY) shows at 13.46 g/100 g d.w. After three repeated tests, the average RS content was 13.44 g/100 g d.w. with a small relative error (RE = 0.42). The actual value was close to the theoretical value, indicating that the optimization results were reliable and practical.

US as a sustainable processing technique has the potential to produce RS with desirable properties and mechanical performance. Modification at different US processing temperatures can achieve functional properties not found in untreated starches, which may have specific applications in the food industry.

### 3.4. Morphological Characterization of Ultrasound-Modified Rice Bran Resistant Starch

**Figure 3:**
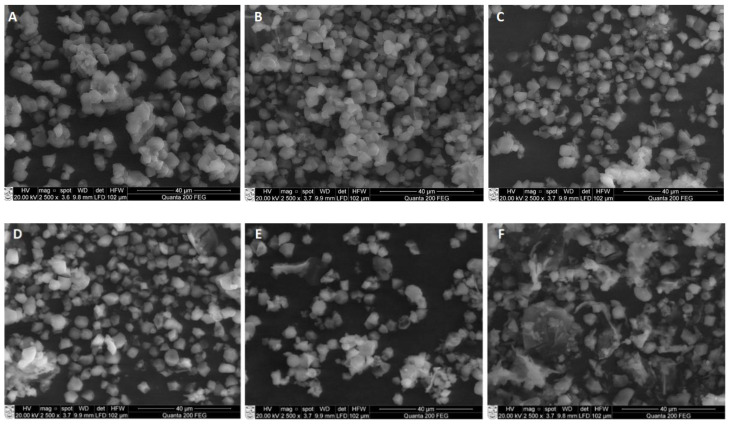
SEM images of native rice starch (**A**), resistant starch isolated from RB untreated (**B**) and ultrasonicated (1.8 W/cm^2^, 20 min) at 40 °C (**C**), 50 °C (**D**), 60 °C (**E**), and 70 °C (**F**) temperatures (magnification ×2500).

Scanning electron micrographs (SEM) of RS isolated from sonicated samples at different temperatures of RB are presented in Figure 3. The micrographs of the native rice starch and untreated RS (RS_UN_) showed typical irregular-shaped small-size granules (Figure 3A,B), corroborating the structure reported by Ashwar et al. [10] and Yang et al. [19]. The changes in the RS granule morphology became more visible when US was applied.

Ultrasonication for 20 min at 40 °C already induced the disintegration of the RS_40C_ granules and the formation of crushed particles (Figure 3C), while the granules of RS_50C_ (Figure 3D) already showed visible differences in shape and size (higher amount of crushed particles) compared to those treated at 40 °C. Elevation of the temperature to 60 °C resulted in an aggregation of small granules (Figure 3E); the formation of a film-like structure was noticed at a temperature of 70 °C (Figure 3F).

Similar tendencies were reported by Noor et al. [33], who indicated a notable difference in the microstructure between RS2 samples of US-treated and untreated lotus stem starch. As Sujka [17] reported, US processing (20 kHz, 170 W, 20 °C, 30 min) affected the average diameter and pore size distribution in rice, corn, wheat, and potato starches. According to Ding et al. [30], the morphological characteristics of retrograded starch (RS3) changed due to high-power US treatment (20 kHz, 100–600 W, 30 min), resulting in a more compact block-shaped structure.

In our study, the increase in the US temperature from 50 to 70 °C tended to a more intensive disruption of larger starch granules and the aggregation of small granules due to the combined effect of the US cavitation and temperature (Figure 3E–G). According to the literature, US cavitation induces high-frequency vibrations, which can break the rice starch and cause a substantial deformation of the granule structure at the starch gelatinization temperature (62–79 °C) [34]. Based on these observations, the change in the pasting behavior is explained in terms of the solubilization of the swollen starch granules and starch aggregates induced by sonication, as was reported by Zuo et al. [35]. Overall, our study showed the visible fragmentation of starch particles of RS isolated from US-treated RB material.

### 3.5. Crystallinity of Rice Resistant Starches

**Figure 4:**
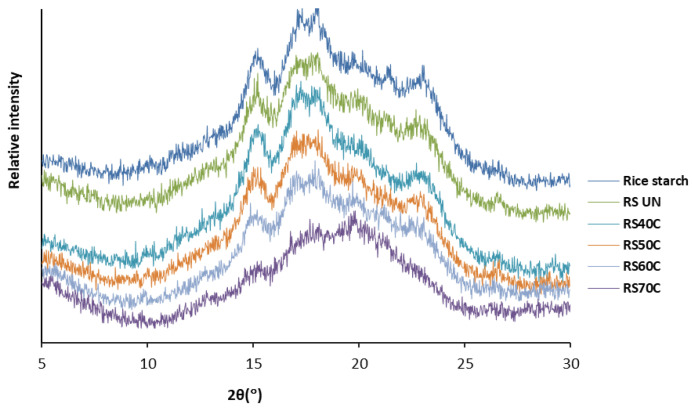
X-ray diffraction patterns of native rice starch and resistant starches (RS) isolated from rice bran that was untreated (UN) and ultrasonicated (1.8 W/cm^2^, 20 min) at different temperatures.

The results of X-ray diffraction (Figure 4) indicate that the untreated RS had the typical A-type crystallinity pattern (corresponding to tight-packed amylopectin double helices) with the characteristic peaks at diffraction angles (2θ) of 15°, 16.9°, 17.9°, a weak peak at 20° 2θ, and an additional peak at 23° 2θ, as is similar to the observations of other researchers [18,36].

According to the literature, the width of the peaks is assigned to the size of the crystallites: a discrete peak represents a higher crystallinity of the starch [25], while a broad diffraction peak indicates lower crystallinity and an amorphous character [37].

Ultrasonication under mild conditions (40 °C) resulted in a minor decrease in crystallinity at 20° and 23° 2θ, a reduced peak sharpness at 15°, and a doublet at 16.9° and 17.9° 2θ compared to the starches isolated from the untreated RB (RS_UN_ sample). The RS samples obtained after sonication at higher temperatures already showed lower crystallinity peaks. With a temperature of 70 °C, the disappearance of peaks at 15° and 23° 2θ, and a doublet at 16.9° and 17.9° 2θ was noticed.

The higher crystallinity of the native rice starch (26.31%) and RS_UN_ (23.85%) (Table 2) implies a stronger interaction between the double helices within the crystalline regions [37]. Similar to maize starch [14], ultrasonication at temperatures lower than the gelatinization temperature did not cause polymorphic changes in the rice RS diffraction pattern (A-type), but a reduction in the degree of crystallinity (up to 17.95–18.36%) was observed. Sonication at temperatures of 60 and 70 °C strongly reduced the degree of crystallinity to 14.40% and 4.43%, respectively.

Jiranuntakul et al. [38] also reported that the crystalline kind of starch in rice with an A-type diffraction design remains unaltered after heat-moisture treatment, with an average crystallite size varying from 4 to 20 nm.

Reduced crystallinity was reported for US treated waxy corn starch [17], rice starch [18], potato starch [39], and oat starch [40], and Noor et al. [33] also reported a decreased crystallinity and increased amorphous character of RS from lotus stem after 35 min of sonication (20 kHz, 100–400 W) under cold conditions. According to BeMiller and Huber [41], changes in the amorphous and crystalline regions, breaking, and destroying the double-helical order during mechanical treatment of starch were detected.

### 3.6. Chemical Structure Characterization

**Figure 5:**
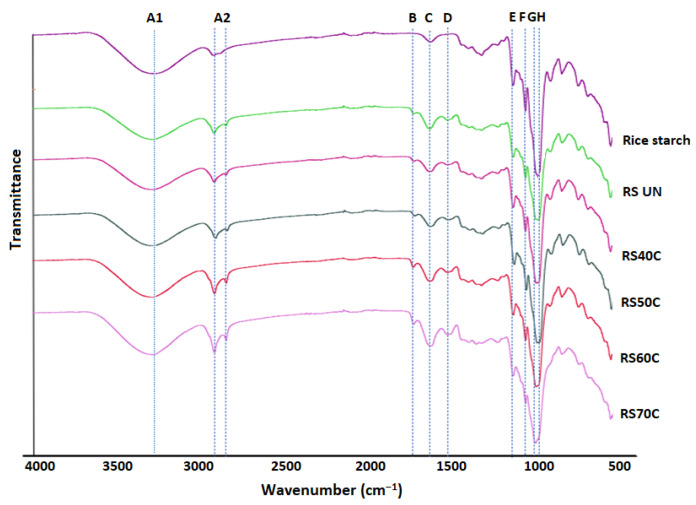
FT-IR spectra of native rice starch and resistant starches (RS) isolated from rice bran (RB) that was untreated (UN) and ultrasonicated (1.8 W/cm^2^, 20 min) at different temperatures.

The FT-IR spectra of the rice starches are displayed in Figure 5. The chemical structure of the starch granules was analyzed after ultrasonication for 20 min at temperatures of 40–70 °C.

The intensity of absorption at 995 cm^−1^ (C–O stretching) and 1149 cm^−1^ (peak E; C–O–C asymmetric stretching), and the intensity of the peak at 1336 cm^−1^, corresponding to C–H symmetric bending [37], was relatively stronger for the native rice starch and RS_UN_ than for the ultrasonicated RS. The RS samples showed C–H stretching at 2926 cm^−1^ (peak A2) and at 1646 cm^−1^ (peak C; C–O bending associated with –OH group) that may be due to the picking of amylose helices during the combined US and thermal treatment.

The stretching at 1514 cm^−1^ (peak D) could be assigned to the asymmetrical stretching of the carboxylate group (–COO). In addition, the bonding to the carbonyl group (C=O) displayed an absorption peak at the 1720 cm^−1^ (peak B) region as a result of a possible polysaccharide oxidation process, which was more noticeable after US treatment. The bands at 999 cm^−1^ (peak H) and 1076 cm^−1^ (peak F) characterize the crystalline structure, and the band at 1016 cm^−1^ (peak G) was attributed to the amorphous structure of the starch [33].

The observed differences in the FT-IR spectra of the RS samples may indicate a greater structural disorganization of the starch macromolecules, and the possible conformational and chemical changes (an increase in amylose content, hydrolysis, or oxidation) as a result of the action of the physical treatment and temperature [42]. The peaks H and F of the native rice starch exhibited the highest absorption, while in the case of the RS samples, the corresponding peaks were of lower intensity. The intensities of the latter bands of the US-treated RS decreased with increasing sonication temperature. The maximum intensity at peak G was reached for the RS samples ultrasonicated at temperatures of 60 and 70 °C, signifying a decreased crystallinity and increased amorphous character.

The results were similar to those reported by Young et al. [18], who reported the stretching of C–OH, C–C, and C–H groups in the case of rice starch modified by US (22 kHz, 25 °C, 20 min), and also with Ma et al. [37], who reported similar alteration of the crystalline and amorphous areas of RS isolated from Laird lentils by thermal treatment. Noor et al. [33] showed a decrease in absorbance at 995 and 1047 cm^−1^, indicating a decreased crystallinity of RS from lotus stem due to US exposure.

In the case of amylose, the RS samples isolated from the US-treated RB had significantly higher (*p <* 0.05) contents of amylose (37.20–53.10%) than the untreated RS (Table 2). An increase of 46.2% of the amylose content was already determined when US was applied for 20 min at 40 °C; an average two-fold increase was observed with increasing sonication temperature at 50–60 °C compared to the RS_UN_ sample (25.62%).

The present results were in line with the study of Falsafi et al. [40], who reported that sonication increased the content of apparent amylose in oat starch, suggesting the depolymerization of amylopectin. US cavitation increased the amylose content in potato, mung bean, sago, and maize starches, indicating the decomposition of the molecular structures and formation of linear amylose fragments [43].

### 3.7. Pasting Properties

**Figure 6:**
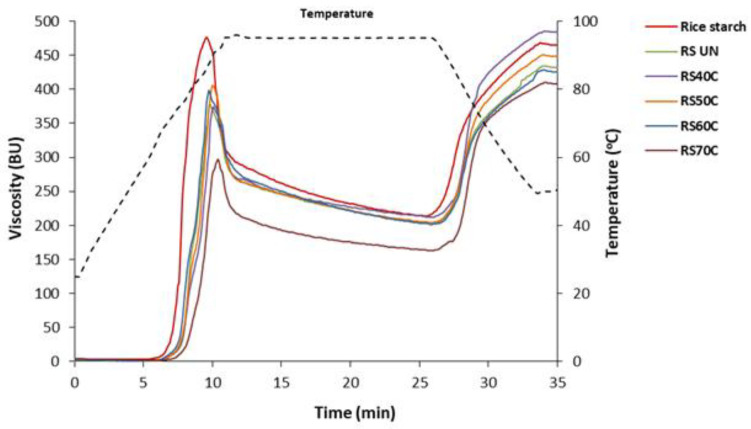
Pasting curves of native rice starch and resistant starches (RS) isolated from rice bran that was untreated (UN) and ultrasonicated (1.8 W/cm^2^, 20 min) at different temperatures. BU: Brabender units.

The pasting curve profiles of the US-treated RS samples were similar to that of the untreated RS (Figure 6), indicating that sonication had no significant influence on the profile shape, compatible with the studies by Yang et al. [18] and Bian and Chung [44].

In the case of the pasting properties (Table 3), the native rice starch had the lowest pasting temperature (67.90 °C) and the highest peak viscosity (474.02 BU), while the RS_UN_ showed a significantly higher (*p* < 0.05) pasting temperature (72.95 °C) and lower peak viscosity (370.63 BU) due to the decrease in the crystalline/amorphous ratio and the double helix content of starch that occurred during the milling of the rice grain [45]. The specific viscosity (η_s_) of the RS slurry increased with a 40 °C US temperature and decreased at temperatures between 50 and 70 °C.

Ultrasonication at 40 °C slightly increased the pasting temperature of the RS_40C_ paste and reduced the peak viscosity compared to the RS_UN_ paste. The final viscosity value of the RS increased significantly at US temperatures of 40–50 °C but decreased compared to the untreated RS due to the disruption and dissolution of the starch granules. This indicates that US-assisted treatment increased the mechanical and thermal stability of the rice RS pastes and caused a higher tendency of the RS granules to retrograde.

With temperatures of 50–60 °C, the RS pastes exhibited a slightly higher peak viscosity (*p* < 0.05) compared to the RS_40C_ with no significant effect (*p* ≥ 0.05) on pasting temperature (Table 3). The RS_70C_ that was isolated after sonication at 70 °C had the highest pasting temperature (75.07 °C) but a strongly reduced peak viscosity (298.51 BU), indicating that the starch granules swelled slowly.

This can be explained by the induced intermolecular interactions, making this starch more resistant to thermal treatment. The percentage content of amylose increased, possibly due to the destruction of starch polysaccharides. The branched structure of amylopectin macromolecules were broken down, depending on the ultrasound intensity, while amylose formed a more compact structure with greater intermolecular interactions, i.e., additional hydrogen bonds due to the increased content of carbonyl and carboxylate groups (Figure 5), increasing the gelatinization temperature of the starch granules. At high US temperatures, the maximum viscosity of potentially damaged macromolecules decreased, which characterizes the interaction of maximally swollen particles (the smaller the particles, the lower the viscosity). In this case, the final viscosity was also lower due to the destruction of the starch polysaccharides and smaller particles, as was confirmed by the higher amylose content and the additional B and D peaks appearing in the FT-IR spectrum. During gelatinization, all starch became amorphous, so there was no significant difference in the profile of the pasting curves. The profile depended more on the shape, size, intermolecular interactions (chemical composition), and molecular weight of the starch granules.

The obtained results agreed with other studies reporting that US treatment reduces the peak viscosity of rice and waxy corn starches [17,34]. The physical damage of starch granules reduces the paste viscosity due to increased water penetration and hydration [46]. For starch, mechanical pre-treatment reduces the granule size and increases the content of the amorphous phase, making it possible to alter the hydrating and paste-forming properties of modified starch [47]. Additionally, sonication, initiating the breakage of hydrogen bonds, thereby reducing the interaction between the starch granules, leads to a viscosity reduction, as could be explained by the profiles of the pasting curves [20].

### 3.8. Technological Properties

The technological properties of the RS isolated from the untreated and US-treated RB material are presented in Table 4. The results showed that sonication significantly (*p *< 0.05) improved the oil (OAC) and water (WAC) absorption capacities, swelling power (SP), solubility (WS), and reduced the least-gelation concentration (LGC) of the RS compared to the untreated sample.

Among the samples, the sonicated RS absorbed the highest amounts of water and oil (5.91–7.54 g/g and 3.08–3.49 g/g, respectively) compared to the untreated RS (4.58 and 2.70 g/g, respectively) and native rice starch (4.30 and 2.37 g/g, respectively). The SP and WS of the sonicated RS samples increased significantly (*p* < 0.05) with increasing US temperature compared to the RS_UN_.

The LGC can be related to the particle size; native rice starch granules form the firmest gel (after retrogradation) at the lowest concentration, while for RS_70C_, the lowest LGC can be explained by the involvement of amylose macromolecules in retrogradation, possibly strengthening RS gels [48]. The native rice starch contained a higher proportion of long-chain amylose, which is less soluble (Table 4).

The increased solubility of the RS samples isolated from the US-treated RB could be explained by thermal/ultrasound disruption, inducing depolymerization and degradation of amylose and amylopectin [48], thus leading to fragments of shorter branched chains, a rise in amylose content, and damage generation on the surface of the starch particles, as was confirmed by SEM.

According to the literature, US cavitation can initiate the degradation of chains between amylopectin molecules, contributing to strengthened linear fractions that can influence the increase in swelling capacity and solubility of starch granules [38]. When the US treatment was performed longer or at higher temperatures, the internal ordered structure of the starch granules changed to a disintegrated form owing to the absorption of water by amylopectin. The degree of swelling is an indicative characteristic of amylopectin, and the ultrasound temperature can lead to a steady loss of crystallinity in amylopectin molecules, thus reducing the viscosity. According to Wang et al. [49], a loss of crystallinity in amylopectin was a result of the US-induced disintegration of potato starch granules. The study by Ding et al. [50] showed that the swelling and solubility of retrograded corn starch after sonication was also improved in comparison to native starch, indicating that the crystalline structure was damaged due to the changes in the interaction between chains within the crystalline and amorphous regions of the starch granule.

## 4. Conclusions

This study showed that US treatment induced the structural disorganization of rice bran resistant starch, changing its physicochemical characteristics and influencing its mechanical preferences and hydration properties. Modification by US processing achieved functional properties not found in untreated starches, which may have specific applications in the food industry. US treatment at different temperatures (40–70 °C) affected the chemical structure, reduced the crystallinity of the RS from 23.85% to between 18.37% and 4.43%, and increased the mechanical and thermal stability of the RS pastes, indicating a higher tendency of the RS granules to retrograde. The pasting results clearly showed that the most viscous paste formed in the case of the RS_40C_ sample, indicating a strong interaction between the colloidal particles and soluble polysaccharide molecules. This finding could partly explain the enhanced content of such starch and its resistance to digestive enzymes. The US processing significantly (*p* < 0.05) improved the oil and water absorption capacities, swelling power, solubility, and gelation properties.

So far, there has been no report on the improvement of RS content through US treatment of rice-milling by-products. The data evaluation indicated a significant effect (*p* < 0.05) of three studied factors (US temperature, time, and power). The regression analysis confirmed that 97% of the data could be described by the obtained mathematical model. Through the optimization using the RSM, optimal conditions for rice bran US treatment were obtained: a time of 18 min, a temperature of 40.2 °C, and a US power of 1.8 W/cm^2^; under optimized conditions, the predicted RS increment was 13.46 g/100 g d.w.

The technological concept adapted in this study differs from the idea of using pure starch as the raw material to increase the RS content. The raw material used in this study was rice bran, which excludes the difficulty of drying the slurry of starch in the following technological stages. US treatment, resulting in a significant increment of resistant starch in the rice bran matrix, thereby increases its potential to be used as a functional bio-based component for food.

## Figures and Tables

**Table 1:** Experimental design and experimental results.

Experiment Number	Processing Variables			dY ^a^ (g/100 g d.w.)		
	*t* (min)	*T* (°C)	*P* (W/cm^2^)	Experimental	Predicted	RE (%)
Low level (−1)	15	30	1.3			
Medium level (0)	20	40	1.5			
High level (+1)	25	50	1.8			
1	1	1	1	2.02 ± 0.08	2.00	0.85
2	1	−1	−1	1.51 ± 0.04	1.53	−1.13
3	−1	1	−1	6.98 ± 0.13	7.00	−0.28
4	1	−1	1	3.82 ± 0.07	3.80	0.46
5	−1	1	1	8.23 ± 0.18	8.21	0.30
6	1	1	−1	1.09 ± 0.02	1.11	−1.56
7	−1	−1	−1	4.42 ± 0.09	4.44	−0.44
8	−1	−1	1	5.94 ± 0.13	5.92	0.42
9	−1	0	1	12.34 ± 0.18	12.48	−1.12
10	−1	0	−1	10.32 ± 0.21	10.28	0.43
11	1	0	1	5.22 ± 0.10	5.26	−0.85
12	1	0	−1	4.21 ± 0.07	4.18	0.83
13	0	1	0	4.87 ± 0.08	4.83	0.87
14	0	−1	0	5.89 ± 0.11	5.93	−0.71
15	0	0	−1	10.75 ± 0.12	10.69	0.60
16	0	0	1	12.72 ± 0.04	12.61	0.84
17	1	0	0	4.76 ± 0.08	4.72	0.85
18	−1	0	0	11.34 ± 0.23	11.38	−0.35
19	0	0	0	11.72 ± 0.12	11.65	0.60
20	0	0	0	11.61 ± 0.19	11.65	−0.34
21	0	0	0	11.65 ± 0.14	11.65	0.00

^a^ dY: resistant starch content; *T*: temperature; *t*: time; *P*: power; RE: relative error.

**Table 2:** Amylose content and degree of crystallinity (%) of native rice starch and resistant starches (RS) isolated from rice bran that was untreated and sonicated at different temperatures.

Starch Samples	Amylose (%)	Crystallinity (%)	X-ray Diffraction Peak Intensity (r.u.)
Rice starch	25.35 ^d^	26.31 ^a^	2677 ^a^
RS_UN_	25.62 ^d^	23.85 ^b^	2663 ^a^
RS_40C_	37.47 ^c^	18.36 ^c^	2568 ^b^
RS_50C_	53.10 ^a^	17.95 ^c^	2385 ^c^
RS_60C_	51.56 ^ab^	14.40 ^d^	2325 ^c^
RS_70C_	49.97 ^b^	4.02 ^e^	1098 ^d^

Data with different superscript letters within the column represent significant differences (*p* < 0.05). RS_UN_: resistant starch isolated from untreated RB; RS_40C_, RS_50C_, RS_60C_, and RS_70C_: resistant starches isolated from RB sonicated at 40–70 °C.

**Table 3:** Pasting properties and specific viscosity (η_s_) of commercial rice starch and resistant starches (RS) isolated from untreated and sonicated rice bran.

Starch Samples	Pasting Temp. (°C)	Peak Viscosity ^a^	Final Viscosity ^a^	ηs(mPa·s)	LGC (%)
Rice starch	67.90 ^d^	474.02 ^a^	467.27 ^a^	4.38 ^b^	8
RS_UN_	72.95 ^b^	382.14 ^bc^	428.06 ^c^	4.27 ^b^	14
RS_40C_	73.47 ^b^	370.63 ^c^	485.34 ^a^	5.78 ^a^	12
RS_50C_	73.93 ^b^	403.74 ^b^	451.62 ^ab^	3.62 ^c^	10
RS_60C_	71.82 ^bc^	398.32 ^b^	426.18 ^c^	3.09 ^d^	10
RS_70C_	75.07 ^a^	298.51 ^d^	408.37 ^d^	2.86 ^e^	8

Data with different superscript letters within the column represent significant differences (*p* < 0.05). RS_UN_: resistant starch isolated from untreated RB; RS_40C_, RS_50C_, RS_60C_, RS_70C_: resistant starches isolated from RB sonicated for 20 min at 40–70 °C temperatures; ^a^ BU: Brabender units.

**Table 4:** Amylose content and technological and mechanical properties of rice starch and resistant starch (RS) isolated from untreated and sonicated rice bran.

Sample	WAC (g/g)	OAC (g/g)	SP (%)	WS (%)
Rice starch	4.30 ^e^	2.37 ^d^	5.22 ^e^	3.64 ^e^
RS_UN_	4.58 ^d^	2.70 ^c^	5.78 ^d^	4.81 ^d^
RS_40C_	5.91 ^b^	3.08 ^b^	6.17 ^c^	6.06 ^c^
RS_50C_	5.59 ^c^	3.15 ^ab^	6.62 ^b^	6.67 ^b^
RS_60C_	6.17 ^b^	3.27 ^a^	9.32 ^a^	16.45 ^a^
RS_70C_	7.54 ^a^	3.49 ^a^	9.58 ^a^	17.01 ^a^

Data with different superscript letters within the column represent significant differences (*p* < 0.05). Samples: RS_UN_: resistant starch from untreated RB; RS_40C_, RS_50C_, RS_60C_, RS_70C_: resistant starch isolated from RB sonicated at an appropriate temperature and time. WAC: water absorption capacity; OAC: oil absorption capacity; SP: swelling power; WS: water solubility; LGC: least gelation concentration.

## Data Availability

Not applicable.

## Supplementary Materials

The following supporting information can be downloaded at: https://www.mdpi.com/article/10.3390/polym14173662/s1, S.1: Chemical analysis; S.2. Determination of technological properties; Figure S1: Rice bran starch and resistant starch isolation and measurement scheme; Table S1: Multiple comparisons between means of different US (1.3 W/cm^2^) temperature groups (significant at *p* < 0.05); Table S2: Multiple comparisons between means of different US (1.3 W/cm^2^) time groups (significant at *p* < 0.05); Table S3: Significant coefficients of quartic model equation in terms of coded factors; Table S4: Analysis of variance of the regression parameters for a quartic model for the response factor. References [51,52,53] are cited in the supplementary materials.
