# Supplementary material for: Optimization of the Sustainable Production of Resistant Starch in Rice Bran and Evaluation of Its Physicochemical and Technological Properties
Source: Polymers (Basel). 2022 Sep 3;14(17):3662. doi: 10.3390/polym14173662 (PMC9460455; doi:10.3390/polym14173662)
Supplement: Supplementary file 1 [file polymers-14-03662-s001.zip › polymers-1885327-supplementary.pdf]

polymers

MDPI

Article

# Optimization of the Sustainable Production of Resistant Starch in Rice Bran and Evaluation of Its Physicochemical and Technological Properties

**Ruta Vaitkeviciene [1*], Joana Bendoraitiene [2], Rimgaile Degutyte [1], Mantas Svazas [1] and Daiva Zadeike [1*]**

## SUPPLEMENTARY MATERIALS

*S.1. Chemical analysis*

Raw material was tested for protein, fibre, fat, and ash contents according to the AACC Official Methods [1]. The crude protein content was determined by the Kjeldahl nitrogen (method 920.152), the percentage of protein was estimated by multiplying the total nitrogen content by a factor of 5.95. Ash was determined by combustion of the sample in a muffle oven at 550 °C (method 942.05). The fat content was determined by Soxhlet extraction method (method 996.01). Total dietary fibre content was determined according to the method 985.29.

*S.2. Determination of technological properties*

*S.2.1. Water and oil absorption capacity*

For water absorption capacity (WAC) and oil absorption capacity (OAC) determination Ahmad et al. [2], 0.5 g of sample was placed in a pre-weighted centrifuge tube and mixed with 6 mL of distilled water or rapeseed oil. After 1 h shaking in a 37°C water bath, the liquid fraction was carefully removed by centrifugation (2,000×*g*, 15 min). The wet residue was weighed (wet weight), and the water/oil holding capacity was expressed as grams of water/oil absorbed by the gram of starch (g/g).

*S.2.2. Water solubility and swelling power*

The measurement of solubility and swelling power of starch was performed according to Li et al. [3] with slight modifications. The sample (0.5 g) of starch ($W$) was mixed with distilled water (5 mL), and was incubated at 30 °C with continuously shaking for 30 min in a water bath. After, it was cooled to room temperature and centrifuged (3,500×*g*, 20 min) ($W_d$). The supernatant was transferred to a glass tube and dried in an oven at 105°C to constant weight as water-soluble starch ($S$). The solubility and swelling power were calculated according to the following equations:

$$\text{Solubility (\%)} = S \times 100 / W \quad (1)$$

$$\text{Swelling power (\%)} = (W_d \times 100) / [W (100 - S)], \quad (2)$$

where $S$ is the weight of dried supernatant (g), $W_d$ – is the weight of wet sediment (g), and $W$ is the sample weight (g). All samples were analysed in triplicate.

*S.3. Experimental section*

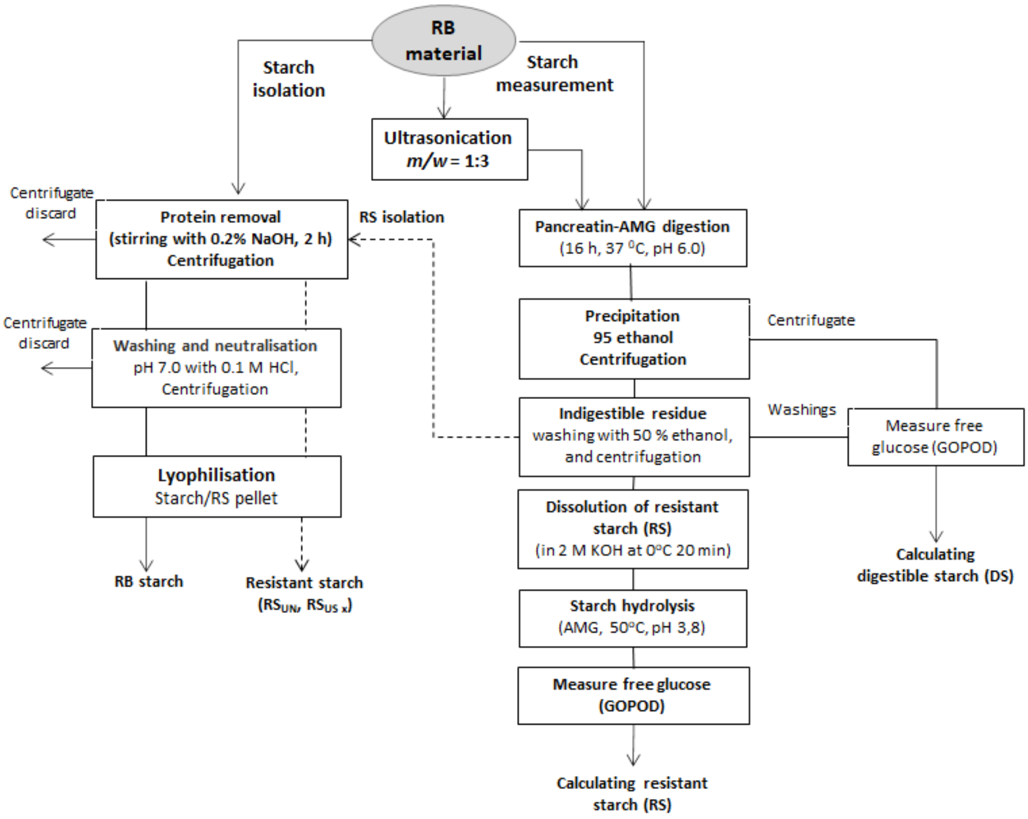


**Figure S1.** Rice bran starch and resistant starch isolation and measurement scheme.

**Table S1.** Multiple comparisons between means of different US (1.3 W/cm$^2$) temperature groups (significant at $p<0.05$).

| | Sum of Squares | df | Mean Square | F | *Sig.* |
|---|---|---|---|---|---|
| 15 min | 616.423 | 4 | 154.106 | 749.007 | 0.0001 |
| 20 min | 614.204 | 4 | 153.551 | 585.090 | 0.0001 |
| 25 min | 321.119 | 4 | 79.137 | 540.531 | 0.0001 |
| 30 min | 68.8417 | 4 | 19.359 | 412.118 | 0.0001 |
| 35 min | 59.785 | 4 | 14.946 | 267.359 | 0.0001 |

**Table S2.** Multiple comparisons between means of different US (1.3 W/cm$^2$) time groups (significant at $p<0.05$).

| | Sum of Squares | df | Mean Square | F | *Sig.* |
|---|---|---|---|---|---|
| 30 °C | 185.611 | 4 | 46.403 | 286.885 | 0.0001 |
| 40 °C | 641.174 | 4 | 160.294 | 560.506 | 0.0001 |
| 50 °C | 372.268 | 4 | 93.067 | 537.090 | 0.0001 |
| 60 °C | 177.368 | 4 | 44.342 | 576.919 | 0.0001 |
| 70 °C | 0.516 | 4 | 0.129 | 21.629 | 0.060 |

**Table S3.** Significant coefficients of quartic model equation in terms of coded factors.

| Factor | Coefficient Estimate | df | Standard Error | 95% CI Low | 95% CI High | VIF |
|---|---|---|---|---|---|---|
| Intercept | 11.65 | 1 | 0.0324 | 11.58 | 11.72 | |
| A-Time | -3.33 | 1 | 0.0463 | -3.43 | -3.23 | 3.00 |
| B-Temp | -0.5519 | 1 | 0.0432 | -0.6496 | -0.4542 | 2.82 |
| C-Power | 0.9644 | 1 | 0.0298 | 0.8971 | 1.03 | 2.62 |
| AB | -0.8837 | 1 | 0.0328 | -0.9578 | -0.8097 | 1.0000 |
| AC | -0.2800 | 1 | 0.0463 | -0.3848 | -0.1752 | 3.00 |
| BC | -0.1419 | 1 | 0.0432 | -0.2396 | -0.0442 | 2.82 |
| $A^2$ | -3.60 | 1 | 0.0565 | -3.73 | -3.47 | 2.41 |
| $B^2$ | -6.27 | 1 | 0.0537 | -6.40 | -6.15 | 2.19 |
| ABC | -0.1387 | 1 | 0.0328 | -0.2128 | -0.0647 | 1.0000 |
| $A^2B$ | 0.8807 | 1 | 0.0542 | 0.7581 | 1.00 | 2.74 |
| $A^2C$ | -0.1399 | 1 | 0.0392 | -0.2287 | -0.0512 | 2.15 |
| $AB^2$ | 1.19 | 1 | 0.0567 | 1.06 | 1.31 | 3.00 |
| $B^2C$ | -0.0930 | 1 | 0.0392 | -0.1817 | -0.0042 | 2.32 |
| $A^2B^2$ | 2.47 | 1 | 0.0781 | 2.30 | 2.65 | 3.94 |
| $A^2BC$ | -0.0643 | 1 | 0.0542 | -0.1869 | 0.0583 | 2.74 |
| $AB^2C$ | 0.3412 | 1 | 0.0567 | 0.2129 | 0.4696 | 3.00 |

**Table S4.** Analysis of variance of the regression parameters for a quartic model for the response factor.

| Source | Sum of Squares | df | Mean Square | F-value | p-value | |
|---|---|---|---|---|---|---|
| **Model** | 389.87 | 16 | 24.37 | 2839.52 | < 0.0001 | significant |
| A-Time | 44.36 | 1 | 44.36 | 5168.84 | < 0.0001 | |
| B-Temp | 1.40 | 1 | 1.40 | 163.30 | < 0.0001 | |
| C-Power | 9.01 | 1 | 9.01 | 1049.64 | < 0.0001 | |
| AB | 6.25 | 1 | 6.25 | 728.10 | < 0.0001 | |
| AC | 0.3136 | 1 | 0.3136 | 36.54 | 0.0002 | |
| BC | 0.0927 | 1 | 0.0927 | 10.80 | 0.0094 | |
| $A^2$ | 34.80 | 1 | 34.80 | 4055.14 | < 0.0001 | |
| $B^2$ | 117.02 | 1 | 117.02 | 13636.80 | < 0.0001 | |
| ABC | 0.1540 | 1 | 0.1540 | 17.95 | 0.0022 | |
| $A^2B$ | 2.27 | 1 | 2.27 | 263.98 | < 0.0001 | |
| $A^2C$ | 0.1092 | 1 | 0.1092 | 12.72 | 0.0061 | |
| $AB^2$ | 3.75 | 1 | 3.75 | 437.29 | < 0.0001 | |
| $B^2C$ | 0,0482 | 1 | 0,0482 | 5,62 | 0,0419 | |
| $A^2B^2$ | 8,59 | 1 | 8,59 | 1001,51 | < 0.0001 | |
| $A^2BC$ | 0,0121 | 1 | 0,0121 | 1,41 | 0,2658 | |
| $AB^2C$ | 0,3105 | 1 | 0,3105 | 36,19 | 0,0002 | |
| **Residual** | 0,0772 | 9 | 0,0086 | | | |
| Lack of Fit | 0,0228 | 1 | 0,0228 | 3,34 | 0,1049 | not significant |
| Pure Error | 0,0545 | 8 | 0,0068 | | | |

| | | | |
|---|---|---|---|
| **Std. Dev.** | 0,0926 | **$R^2$** | 0,9998 |
| **Mean** | 7,78 | **Adjusted $R^2$** | 0,9994 |
| **C.V. %** | 1,19 | **Predicted $R^2$** | 0,9736 |
| | | **Adeq Precision** | 153,6987 |

## References


1. *Approved Methods of the AACC International*, 10th ed.; The Association AACC: Saint Paul, MN, USA, 2000.
2. Ahmad, M.; Gani, A.; Masoodi, F.A.; Rizvi, S.H. Influence of ball milling on the production of starch nanoparticles and its effect on structural, thermal, and functional properties. *Int. J. Biol. Macromol.* **2020**, *151*, 85–91.
3. Li, A.; Gao, Q.; Ward, R. Physicochemical properties and in vitro digestibility of resistant starch from mung bean (*Phaseolus radiatus*). *Starch/Stärke* **2011**, *63*, 171–178.
